# Service Providers' Perspectives: Reducing Intimate Partner Violence in Rural and Northern Regions of Canada

**DOI:** 10.1177/08445621221128857

**Published:** 2022-09-29

**Authors:** Nicole Letourneau, Dawn Lorraine McBride, Sylvia S. Barton, Keira Griggs

**Affiliations:** 1 2129 University of Calgary, Calgary, AB, Canada; 2 4512 University of Lethbridge, Lethbridge, AB, Canada; 3 6727 University of North British Columbia, Prince George, BC, Canada

**Keywords:** domestic violence, community/legal intervention, service providers, sociocultural context, diversity, grounded theory

## Abstract

**Background:**

Intimate partner violence (IPV) persists as a serious challenge, globally, with regions in Central and Northern Canada reporting the highest rates of shelter use to escape abuse, of sexual assault, and of IPV in the country. Despite research into IPV, barriers and gaps exist in understanding what an effective response to IPV in rural and northern communities should look like.

**Methods:**

To enhance this understanding, qualitative interviews and focus groups with a total of 55 participants were conducted with service providers, including shelter services, victims services, the Royal Canadian Mounted Police, counselors, and others (e.g., psychologists). A grounded theory approach was used to analyze data, with findings illustrated in a schematic that conceptualize the challenges service providers experience.

**Results:**

The findings reveal how an IPV environment, characterized by oppression, abuse, and illness, requires transformation into an IPV-free environment, characterized by empowerment, positive social connections, and wellness. As service providers work to influence this transition, they become experts in understanding the sociocultural context, formal services, and informal supports accessible or not for women experiencing IPV. Service providers encourage social media use into service delivery to improve communication; lobby for rural-specific IPV specialists; and recognize isolation as a barrier to seeking out safe shelter and housing, transportation, and economic assistance.

**Conclusion:**

In order to reduce rates of IPV, the results suggest we must support service providers, document service gaps, and maximize policy change and community action based on IPV as it is experienced in rural and northern regions of Canada.

## Background and purpose

Intimate partner violence (IPV) refers to controlling, physically aggressive, psychologically abusive, and sexually coercive behaviors by an intimate partner or ex-partner that results in psychological, physical, or sexual harm (Ellsberg & Heise, 2005; World Health Organization [WHO], 2017). IPV is also known as domestic violence, violence against women, or gender-based violence (Siemieniuk et al., 2013). IPV is a significant global health problem of epidemic proportions and a fundamental violation of human rights. A report on global and regional estimates of violence against women indicates that approximately one in three women worldwide have experienced different forms of violence, with IPV being the most common form of violence (WHO, 2021). Other striking findings include intimate partners committing up to 38% of all murders of women and that variation in the prevalence of violence seen within and between countries highlights the need to address the diverse sociocultural and economic differences that perpetuate societies of violence against women (WHO, 2013). In Canada, a woman is killed by her intimate partner approximately every 6 days (Cross, 2018), and 67% of Canadians know a woman who has experienced physical or sexual abuse (Cross, 2018). Other facts include Indigenous women being killed at a rate six times the rate of non-Indigenous women and that, on any given night, more than 6,000 women and children sleep in shelters because their home is not safe (Cross, 2018).

Despite mounting research in western societies on the prevalence of violence against women, numerous gaps exist, including an understanding of IPV within a Canadian context and the unique needs of women living in rural and northern communities (Tam et al., 2016). Women experiencing IPV in rural and northern regions of Alberta and the adversities they face are less understood. These knowledge gaps about IPV in Alberta require a deeper understanding in order to address an informed community response, specifically related to hardships as a result of living in a rural and northern context (Bailey, 2013). An IPV environment and its circumstances have received far less study compared to research conducted in easier-to-access urban centers (Bailey, 2013). In Alberta, studies have primarily focused on clinics screening of women and measuring of IPV prevalence (Thurston et al., 2006), with some research focused on understanding the experience of women and IPV (De Riviere, 2014; Tutty et al., 2009). Less emphasis has been placed on addressing specific challenges of rural and northern community responses to IPV (Tutty et al., 2006). Thus, the purpose of this article is to address this knowledge gap by focusing on an IPV community response specific to rural and northern regions of Alberta.

### Women's experience of IPV in Canada and Alberta

Less than 30% of cases of IPV are reported to police, and 44% of Canadian women report experiencing physical or sexual assault in an intimate partner relationship in their lifetimes (Cotter, 2021). In Alberta, the annual provincial rate of reported IPV was 9% (Sinha, 2013), and a report from the Alberta Council of Women's Shelters (ACWS, 2019) noted thousands of women are sheltered and provided safety from abuse annually and thousands are turned away due to unavailable shelter space.

Women experience more severe injury due to IPV than men (Burczycka, 2019). Women experience not only physical injuries from IPV, but also long-term, adverse conditions that affect all dimensions of health, including chronic physical, mental, and reproductive health as well as health outcomes specific to pregnancy (Guruge, 2012). At the family level, further complications exist, including an increased financial burden associated with the costs of extended healthcare coverage and traveling to access healthcare services (Statistics Canada, 2017). Children witnessing IPV are negatively affected, psychologically (Tutty et al., 2009), and IPV costs society in terms of healthcare, social services, and court justice system expenditures (Statistics Canada, 2017). In addition, poverty, homelessness, loss of employment, and decreased earnings associated with IPV further negatively impact society (Wells et al., 2012).

## Rural and northern challenges experienced by service providers

Although there is an increased focused on IPV in rural and northern communities, a paucity of research still exists, especially in rural Alberta, where 17% of the 4.5 million provincial population lives (Statistics Canada, 2011, 2022). The term rural can be understood as individuals living outside areas with a population of 1,000 or more (Statistics Canada, 2011), and northern as approximately the upper one third of the Alberta Prairie Province. In terms of understanding women's and service providers’ experiences of IPV specific to these regions, less research exists, justifying an urgent need to explore rural and northern community responses to IPV. Challenges unique to rural and northern regions of Saskatchewan have been highlighted to include frustration related to slow response times, a lack of financial and emotional support for women experiencing IPV, as well as with high staff turnover due to professional burnout (Wuerch et al., 2016). In other studies, gaps identified exist in understanding what an effective response to IPV in rural and northern communities should look like, especially related to hopelessness resulting from limited IPV resources, transportation options, and needed services (Merchant & Whiting, 2015). These authors also highlighted how service providers are pushing back at these forces, with services that are taking shape and gaining traction in certain under-resourced regions, such as victim services, police transportation, and emergency intervention orders (Faller et al., 2018). Nevertheless, more needs to be done to reduce the prevalence of IPV in rural and northern communities, as service providers require an understanding of the complexity associated with geographical challenges, including diverse sociocultural, legal, and employment barriers.

The intent of this study was to increase an understanding of the experience of IPV service providers working in rural and northern regions of Alberta, specifically by examining barriers and gaps that exist in understanding effective responses to IPV. By exploring experiences of local leadership in these communities and by examining service provider perspectives, a more comprehensive understanding of the service barriers and unique needs that exist for women in Alberta was identified. As service providers work to influence a transition from an IPV environment to an IPV-free environment, they revealed expertise in explicating an understanding of the diverse sociocultural context of IPV as well as the formal services accessible and informal supports available or not for women experiencing IPV. The study outcomes will contribute to efforts directed at further reducing rates of IPV in these regions by supporting service providers better, documenting service gaps more clearly, and maximizing policy change and community action related to IPV as it is experienced in rural and northern regions of Canada.

## Methods and procedures

### Data collection

This study was part of a multiyear, multisite Social Sciences Humanities Research Council's Community-University Research Alliance grant titled “Rural and Northern Community Response to Intimate Partner Violence” The project was conducted across geographical communities located in rural and northern regions of Alberta, Saskatchewan, Manitoba, and the Northwest Territories. During the first phase of the project, these regions were mapped using geographical information systems methods to understand the location of available IPV-related services in all rural and northern regions and the prevalence of IPV incidents in these areas. During the subsequent phases, this information informed the selection of regions across rural and Northern Alberta, where qualitative interviews and focus groups were then be conducted with service providers between 2016-and 2017. Here, we focus on the identified barriers and gaps that exist in understanding an effective response to IPV in rural and Northern Alberta.

Following approval from University Health Research Ethics Review Boards, participant recruitment and data collection were completed in 10 geographically distanced communities representing regions of rural and Northern Alberta. In collaboration with our community partners, the authors selected communities best representing rural and Northern Alberta, settling on a total of 10 centers nine with ‘small’ populations less than 30,000, and with ‘medium’ population of less than 100,000 (Statistics Canada, 2011). Of note is that women living in these areas accessed services mostly from their respective rural, rather than urban, centers; thus, we felt confident in collecting data that could identify the barriers and gaps that exist in understanding what an effective response to IPV in rural and Northern Alberta should look like.

#### Recruitment

Qualitative research using grounded theory methodology guided the study, with purposive and snowball sampling informing the selection of participants (Creswell, 2017). These participants were experts in the field of knowledge and practice related to managing the service needs and gaps for women in their areas. Participants included employed Royal Mounted Police (RCMP) officers (*n* *=* 10), shelter services employees (*n* *=* 10), and victim services employees (*n* *=* 10). In addition, participants in three focus groups included RCMP officers (*n* *=* 2), administrators of government services programs (*n* *=* 4), addictions counsellors (*n* *=* 2), social workers (*n* *=* 5), nurses (*n* *=* 3), shelter services employees (*n* *=* 4), victim services employees (*n* *=* 3), and psychologists (*n* *=* 2). Permission was obtained to interview the RCMP officers as required through regional RCMP offices; all other participants were contacted directly. All participants were over 18 years of age and had over 10 years of experience assisting women experiencing IPV in rural and Northern Alberta.

#### Interviews

Qualitative semi-structured telephone interviews and open-ended focus group interviews were conducted with 55 service providers from 10 rural and Northern communities in Alberta. The telephone interviews were conducted due to financial, time, and geographical constraints, each lasting 1.5 h. All focus groups were conducted face to face in three communities, each lasting 2 h. The interview guide facilitated participants to reflect on and respond to the following questions:

- What are the unique needs of women who experience IPV living in rural and Northern regions of Alberta?
- What are the barriers that exist in meeting these needs?
- How do we create and sustain nonviolent communities in Alberta?

Participants were first telephoned and then emailed the study information letter and consent form to invite interest and to secure their commitment. The interviews and focus groups were conducted by an experienced academic researcher and research program coordinator following signed consent that assured confidentiality, privacy, and voluntary participation. Participants had the option of not answering questions or of exiting the study at any time. The telephone interviews and face-to-face focus groups were digitally recorded. All identifying information was removed from the transcripts, and the names on consent forms were kept separate from the interview data.

### Data analysis

A grounded theory approach (Corbin & Strauss, 2008) guided each stage of data analyses that began after all interviews were transcribed. A research team of academic and community partners from the Alberta site started with open coding to form categories of information about the data from each transcript. Open coding is a way to describe what is occurring in the data and resulted in the formation of overarching concepts. Axial coding was then undertaken to explain how these concepts that formed explanations were connected or related to other codes or categories. Selective coding followed, resulting in the development of an overarching or central theme that emerged from the data, describing and explaining the phenomenon in Alberta. The phenomenon of experiences of IPV in Alberta was then developed into a pictorial diagram (Barton et al., 2015). This was useful in that it facilitated the assembling of data into a visual representation of the analysis in order to show the social, historical, and economic conditions influencing the phenomenon (Creswell, 2017). The study allowed for the discovery of a theoretical framework from data generated from the perspectives of participants (Moghaddam, 2006), resulting in a final step of development of a central schematic concept of transitions from an IPV environment to an IPV-free environment. The central concept provides a theoretical framework to guide future action and research (Creswell, 2017). The analysis and schematic were discussed through regional team meetings for agreement and consensus to ensure rigor, and the findings were presented through local, national, and international invited presentations and conferences to seek feedback, all of which further substantiated the validity and usefulness of the theoretical framework. Finally, the trustworthiness of findings was considered via assessment of the criteria of credibility, transferability, dependability and confirmability (Lincoln & Guba, 1985; Nowell et al., 2017).

## Results

Service providers discussed a deep understanding of the barriers (e.g., underuse of mobile communication technology) and unique needs (e.g., lack of public transportation) they encountered when aiding women experiencing IPV in rural and northern regions of Alberta. They also explained the overarching pivotal themes (e.g., context, formal services, informal supports, and protective factors) that can be manipulated to create and sustain nonviolent communities. Throughout their discussions, service providers emphasized transitions and how they helped women experiencing IPV to shift their reality and circumstances by harnessing increased sociocultural contextual awareness (e.g., education and counselling needed by different cultural groups), to take advantage of more formal services (e.g., safe shelter and legal services) and to leverage as much informal support as possible (e.g., originating from family, friends, and community members). This idea of transition at the core of their discussions was proactive, action-oriented, and collaborative and could result in substantial life-changing events for women. It presented many challenges for service providers to alter an IPV environment in partnership with women, characterized by its cultural norms reinforcing IPV; existence of closed communities; poor communication; physical, psychological and sexual abuse; controlling behavior and intimidation toward women; use of emotional, spiritual, and religious abuse; limited ability to enjoy life; limited social connections/isolation; and poor health. Yet, they were determined and committed in their work toward creating and sustaining nonviolent communities in rural and Northern Alberta, characterized by the existence of healthy communities; gender equality; positive social connections; ability to meet basic needs with or without a partner; wellness and health; recreation opportunities; community involvement; transparency; positive community leaders; prosperity; and rich cultural experiences.

**Figure 1.**
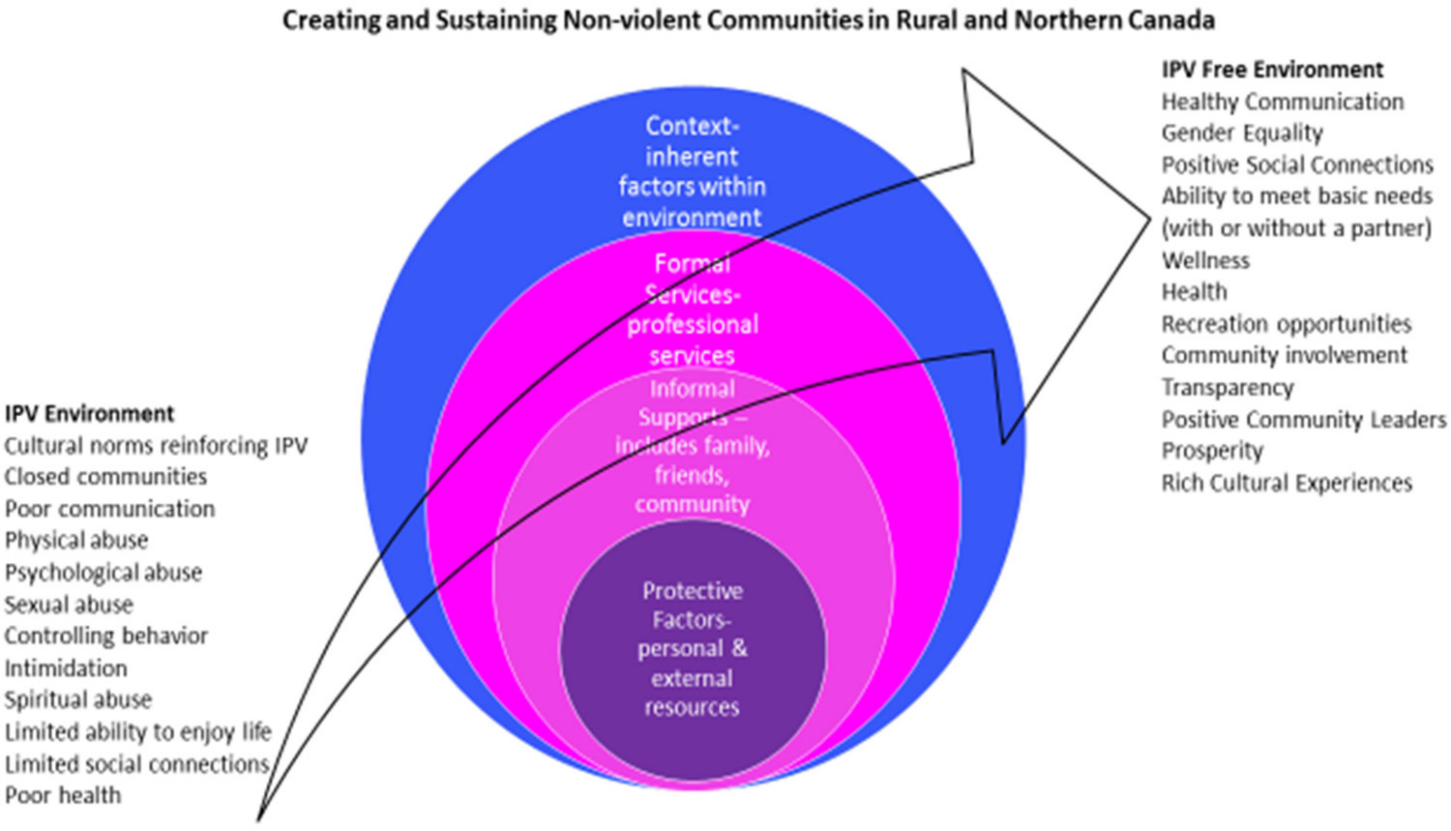
Conceptualization of the IPV challenges service providers experience in Rural and Northern Alberta Communities.

A schematic was developed to explain how service providers understand rural and northern community response to IPV in Alberta (Figure 1). The schematic depicts a set of inner circles that are surrounded by barriers and unique needs experienced by women, all within an environment in which IPV occurs and ranges to an environment that is IPV free. For transitions to occur, they must extend through the four circles that represent overarching themes in which service providers acquire an understanding of women's experience of IPV. They are nested categories from which service providers address: (a) a circle of context (e.g., inherent factors within an IPV environment such as sociocultural and economic differences adversely affecting access to needed resources), (b) a circle of formal services (e.g., need for professional services delivered through an integrated case management approach), (c) a circle of informal supports (e.g., connections with family, friends and community members that positively envelop women during periods of IPV recognition, crisis and intervention), and (d) a circle of protective factors (e.g., leveraging the ability to activate personal and external resources based on values that include zero tolerance for IPV and recognition that the perpetrator must receive treatment as an abuser). The schematic further illustrates how the four circle categories form a theoretical framework for understanding the complex provision and implementation of services and how women experiencing IPV in rural Alberta need to be supported. For instance, the theoretical framework gives a picture of how service providers recognize an IPV environment in rural and Northern Alberta (challenges for women include an inability to access affordable housing independently, lack of childcare that prevents women from seeking support and employment and from attending medical appointments, and lack of community resources in close proximity due to geographic isolation). Central to the schematic is the capacity to cut across these rural and northern adversities and turn them into advantages. Service providers specified that lack of movement for women existing in an IPV environment was emotionally troubling, yet it was from this vantage point of moving away from an IPV environment that practical plans and interventions could be implemented. The following results provide more details for each of the overarching categories and what service providers experience as they work to remove barriers and address unique needs of women experiencing IPV in rural and Northern Alberta.

### Context

In understanding a community response, contextual factors within the IPV environment reveal the intersections of how community diversity, environment, and geography are experienced by service providers. Through an urgent desire to improve the response, service providers reveal a vision of change that is informing a more effective and comprehensive delivery approach. However, for change to be successful, many participants indicated recommendations will benefit only to the extent that community diversity is understood. As a lens through which to look, participants shared an understanding of IPV that encompasses diverse cultural, socioeconomic, and relationship spectra. In terms of diversity, communities included Indigenous women; partners of men temporarily employed in the coal, oil, and gas industry; women living on farms; women within immigrant and refugee communities; and members of the lesbian, gay, bisexual, transsexual, and queer (LGBTQ) community. In the sections that follow, excerpts from participants are cited using the terms Interviewee or Focus Group Participant.

#### Indigenous women

Women from diverse backgrounds present service providers with unique challenges. For example, in Indigenous communities, finances may be controlled by the chief and council, which has implications for accessing housing and other resources. Historical trauma experienced by Indigenous people compounds the complexity of IPV issues, such as the intergenerational cycle of IPV, normalized behaviors associated with IPV, tolerance of IPV, isolation, and a closed community mindset toward IPV services and providers. Indigenous communities may have less access to police services and less police protection than non-Indigenous communities. Service providers were frustrated with the lack of community infrastructure in some Indigenous communities, especially the lack of public transportation, a legal court system, and childcare services. The only form of transportation may be a medical van, which may be an option for getting to a shelter in another region. While some communities have been able to create some infrastructure, service providers reported women are often hesitant to use these services due to concerns regarding their privacy and confidentiality. One participant explained that there could be up to 90% of Indigenous women temporarily housed at women's shelters, with many being young and under 30 years of age. One victim services provider noted,

Indigenous women we see coming from rural areas may have three children or more, may be on welfare. They can have many obstacles set in front of them related to the generational cycle of IPV, normalized behaviors associated with IPV, tolerance for IPV, experience of extreme isolation and lack of support from community members. (Focus Group Participant)

#### Women as part of a transient community

For service providers, a common theme was a discussion of women as part of a transient population, who were partners of men temporarily employed in the industrial sectors of coal, oil, and gas. Although they lived in a resource-rich community, they had limited access to social resources. For example, there may be no psychiatrist, only psychologists, as it is challenging to obtain psychiatric help for Albertans who do not live near a city. Boom and bust cycles of employment, with fluctuating socioeconomic status and material wealth, were viewed as influencing IPV incidences and sexual assaults. During a boom phase, oil industry wages could be high and workers young. If a woman ended up in an abusive relationship, she may not leave her violent partner due to her reliance on his finances in addition to a lack of affordable housing options. Many service providers characterized bust cycles as periods of stress, vulnerability, and helplessness, which from their perspectives resulted in increased need for IPV services.

#### Women living on farms

Many service providers described women living on farms, who may have to contend with traveling large geographical distances between home and IPV services. This dramatically interferes with their prevention and intervention service options. Women living on farms have challenges accessing financial benefits from their land (e.g., cyclical selling of farm produce, machinery, equipment, and land). Service providers noted women on farms believe they are excluded or perceive exclusion from subsidized support programs with a financial qualifier because they own a share of the farm, or no share at all. Such remoteness can prevent women from taking action, whereby social isolation and small group farm cohesion can lead to self-isolation and the secrecy of IPV in order to maintain obligations and reputations. A shelter services participant stated,

We do see farm women occasionally … with a partner sometimes working in the oil patch, so he might be gone for three weeks and back for a week. So she's really running the farm, and if they have horses or cattle, she is the caregiver to the animals. Unless she can make alternate arrangements, it can be hard for her to leave. (Interviewee)

#### Immigrant and refugee women

Filipino, Low-German (Mennonite), Chinese, and Muslim ancestries represent immigrant and refugee women in rural and Northern Alberta communities. Service providers described how women may be responsible for large numbers of children, experiencing physical or mental illness, and dealing with language barriers (e.g., creating a desperate need for translator services). Immigrants and refugees tend to flock to the cities but many groups live in rural areas. Women may be kept isolated, dependent, and rejected from their cultural community when experiencing IPV. Furthermore, new immigrants can be slow to adjust to the rights that women have in Canada, adding to the complexity for service providers to respond to IPV (e.g., separation and divorce may be taboo and cause shunning, may not be registered with immigration). A victim services participant stated,

Cultural value systems vary, their ideology, the cultural aspects of their lives are such that if domestic violence occurs, very few report.… Reporting is a way of helping, but if they’re not going to report culturally, perhaps we need to put in a system that supports them from within versus outside interference, which they don't want? (Interviewee)

#### LGBTQ community

Service providers indicated that members of the LGBTQ communities tend to be invisible in rural communities and shared they have limited awareness of their presence. The LGBTQ community is regarded as less known and all service providers revealed that they could not identify any LGBTQ-specific IPV services or knowledge of what the needs are in regards to IPV. Service providers stated that LGBTQ relationships tend not to be out in the open and in some areas there is known discrimination. As one service provider noted,

LGBT is not as common in rural areas. Everyone knows everyone and so relationships are not out in the open. There are no IPV services for LGBT, specifically, and in some areas discrimination clearly exists. So it's difficult for people to be openly gay or bisexual in rural areas.… There's a lack of knowledge of how services could be tailored to meet their needs but I think there is more acceptance toward LGBT individuals now than 10 years ago. (Focus Group Participant)

### Formal services

#### Provision of safe shelters

All service providers discussed the need for accessible safe places. Certain conditions related to service provider expertise that favored the successful provision and management of formal services, such as shelters, included the ability to integrate long-distance and public transportation options into the service, exhibiting nonjudgmental attitudes and cultural sensitivity during the complex process of how a woman leaves an IPV environment, knowledge of educational options for isolated cultural groups; and understanding of resources for independent living that are separate from the IPV offender's income. Although service providers may want to provide these conditions to all women, they claimed there exists an inequitable delivery of formal services across rural and Northern Alberta and more barriers compared to urban areas.

#### Working together

Service providers discussed the need for promoting awareness of IPV between each other and collectively offering choices to women that led to desirable change. They viewed themselves, for instance, as being well positioned to influence the degree to which women experience feelings of self-blame, low self-esteem, and unworthiness. Service providers expressed a need to reach out to these women in different ways, to offer more emotional support and mentoring, and to instill a sense of hope and bolstered self-esteem. They described the best practice underpinning the provision of formal services was to nurture a partnered relationship with women experiencing IPV. These included service providers instilling positive notions of autonomy, a dedication to addressing IPV, acceptance of a woman's decision to return to the offender, asking for permission to help the abuser, committing to long-term solutions, and activating language translation services. Service providers further revealed examples of how the positive influence of service providers had led to employment positions for women in social agencies, completion of English courses, and participation in programs aimed at increasing social connections.

#### Integrated case management

Service providers shared that integrated case management is the most effective way to ensure the best outcomes for women experiencing IPV in rural and Northern Alberta; in some cases this approach also included how to help the IPV offender. Formal services related to women's shelters, healthcare, mental health, addictions, sexual assault, policing, victimization, legal support, child and family wellbeing, and schools depend on various service providers communicating for the big-picture coordination of multiple individual and family needs. Service providers explained how they connected the dots by sharing information between agencies and communities, determining what services could be offered. The collaboration provided clarity around their professional scope of practice that exists across service providers’ professional disciplines. This approach resulted in more effective team communication that addressed the needs of women experiencing IPV in rural and Northern Alberta (e.g., navigating a different standard of justice in Alberta between northern and rural court systems compared to urban court systems). One social worker noted,

There is effective, long-standing relationships in this group (of integrated case managers) regardless of the differences in mandates. There's an opportunity to influence each other… We do a lot of case conferencing, meetings are set up quickly here, and we can pull the players together and really look at how we can come together in spite of each person doing something different. (Interviewee)

### Informal supports

Informal supports were described by service providers as originating from families, friends, and community members as well as being drawn from cultural, faith-based, and business organizations. These social connections are integral to a woman's capacity to alter the course of IPV over time. Service providers expressed the need for women to be enveloped within trusted social circles and safe places from which shelter provision and crisis support may be received, especially if formal services are not available (e.g., when shelters are full or there is a lack of police response). One shelter service staff member stated, “In rural communities we do not have huge services available to people and that's just economics. Not every community can pay for a victims’ assistance group or a community hub; they just can't” (Focus Group Participant).

#### Social connections

The effects of social connections are far reaching; service providers explained these effects as originating from close social support networks, whereby women were helped to leave the abuse and live independently, complete long-distance calls, access free or affordable childcare, maintain self-sustaining employment, and act on educational opportunities. More distant social support networks were identified as originating from community organizations, churches, and affordable legal services that resulted in, for example, landlords lowering rent or actually pardoning missed rent, banks forgiving a mortgage payment, companies donating large sums of money to food banks, requests for immigration and employment insurance being quickly processed, businesses providing vehicles for relocating a woman and her children, free recreation passes being distributed, and air tickets given to women to stay with extended family members.

#### Quality of rural and northern life

All service providers noted positive and negative associations existed between living in rural and Northern Alberta and the quality of family life. The extent of the influence of this geography on women experiencing IPV was variable. Living in rural and Northern Alberta was certainly viewed as contributing to the recovery of emotional, mental, and physical wellness, but also as an area that was inextricably linked to shifting societal values and beliefs. Service providers expressed that their witnessing of these positive informal supports took the form of people engaging with women experiencing IPV through interpersonal interactions characterized by nonjudgmental attitudes, generosity in the provision of parental support, and random acts of emotional kindness. As one victim services provider stated,

Antelopes shake for 3 days after being chased by lions. Women may shake after this traumatic experience too. So having to meet with an outreach worker and having them affirm their decision to now leave this person and leave the abuse … so they don't flounder. I’m thinking of one particular case where the outreach workers really helped the person to take a different path. (Interviewee)

#### Community values

Service providers offered suggestions for sharing research that revealed a domestic violence situation is unlikely to get better unless the perpetrator gets treatment for the abuse. This includes the importance of instilling family and community values that recognize zero tolerance for IPV, a belief more important than wealth and social status. This community understanding accepts IPV as wrong and immoral. Service providers expressed such community actions as the reporting of IPV to police by community members who witness it, including breaches of conditions by IPV offenders, interrupting the generational passing on of IPV behavior, overcoming fear of IPV, teaching societal human rights from ethical wrongs, instilling a deep sense of IPV as something that should not happen at all, and narrating the belief that asking for help from others regarding IPV is an acceptable action.

#### Gender equality

Service providers highlighted the need for social networks that value gender equality. The nature of IPV and rural and northern geography can inform an understanding of IPV. Gender equality was explained as removing cultural barriers in the seeking of outside support, believing women experiencing IPV who report IPV, dispelling the belief that the use of violence by partners is acceptable and instead that it is a criminal offence, communicating a counter narrative of women as property, not agreeing with the ideology that purports physical violence is acceptable, and accepting women experiencing IPV not as those who have made bad choices, but as those who are not deserving of bad partners.

#### IPV education

Service providers expressed concern regarding the need to be informed about the definition and prevalence of IPV; the IPV cycle; an awareness of the magnitude of risk involved in IPV; the time commitment for court hearings and its effect on family and personal rights; available IPV services; the connection between IPV, alcohol, and drug use; and recognition that external influences such as decreased employment can potentially increase the occurrence of IPV. Service providers viewed themselves in a position to educate, recognizing that community members have the power to influence the response to IPV. They described examples that included participation of members in community justice forums and restorative justice groups; public support for gun license suspension, in light of current IPV homicides; and the promotion of healthy family and community activities that are drug and alcohol free.

### Protective factors

Service providers described protective factors as actions the woman experiencing IPV could enact to alter her situation. These related to the ability to self-assess and alter the course of IPV, knowing personal and autonomous rights, acknowledging the right to build confidence and self-esteem, learning about the cycle of abuse, being able to assess the danger risk and abuse cycle of IPV, and accepting the fact that IPV is not a normal relational circumstance but a crime. Consequently, many service providers discussed the low socioeconomic status of women in rural and Northern Alberta and how it creates barriers for living independently from IPV offenders. Service providers indicated that society tends to place the responsibility on women to leave the home, when it is the perpetrator who should be leaving.

#### Facilitation of problem-solving

Service providers offered suggestions for how they could assist women experiencing IPV to harness protective factors that provided women with knowledge of services and effective ways of accessing them. This would lead to problem solving other related challenges, such as transportation, childcare, addiction treatment, specialized sexual assault services, specialized IPV legal support, and programs for children exposed to IPV, as well as provincial police, victim, and family services. Service providers also indicated that even for women needing shelter, and who appear to be connected to social networks such as family and friends, options may not exist. One shelter service staff member stated,

The first one is, where are they going to go? Yes, we have shelters but is there space in those shelters? [Women also ask,] “How do I get into a shelter? Who should I talk to? Are they going to tell people? Is my partner going to find out before I even get there? And then when I do find a place, how am I going to get myself and the kids there?” (Interviewee)

## Discussion

Service providers reported that important differences exist for women experiencing IPV in rural and Northern Alberta, that go beyond rurality and being situated in a northern region. Similarly, Little (2016) drew attention “to the need to focus on the local and the personal experience of violence and to situate those personal experiences within the evolution and performance of local cultures” (p. 14). The overarching pivotal themes identified in this study included context, formal services, informal supports, and protective factors. They intersected through these thematic categories that service providers experienced as they worked with women to transition from an IPV environment to an IPV-free environment. The context category served to guide deeper understandings of Indigenous women, women as part of a transient community, women living on farms, immigrant and refugee women, and LGBTQ members. The formal services category served to inform the provision of safe shelters, how to work together with women experiencing IPV in rural and Northern Alberta, and an integrated case management approach. The informal supports category served to leverage social connections, quality of rural and northern life, community values, gender equality, and IPV education. The protective factors category served to address the facilitation of problem solving from the perspective of service providers working with women experiencing IPV. A paucity of research exists on sociocultural factors in rural and Northern Alberta that facilitate women to transition away from an IPV environment. This study contributes to IPV research by identifying the intersections of different sociocultural factors that assist individuals (women, men, and service providers) to mitigate and eliminate IPV.

Based on service providers’ responses, the idea of women transitioning is central to facilitating life-changing events related to combating IPV. This is different from other research conducted in rural and northern regions, which identified factors that severely effect women in IPV relationships (Murray et al., 2015; Wuerch et al., 2016). Tutty et al.'s (2006) findings revealed how some rural community members defended IPV offenders, even normalized IPV, and limited the definition of IPV to physical assault only. In addition, traditional values in communities were found to contribute to victim blaming and a lack of understanding of women's IPV experiences. As our study has shown, some of these attitudes appear to have softened but have not been eliminated. Compounding such attitudes is the low socioeconomic status experienced by some women that service providers witness, due to the higher cost of living in rural and Northern Alberta compared to urban areas. This may affect women in two ways. First, it increases the cost of all basic necessities, and, second, it creates numerous barriers for living independently from IPV offenders. As explained by the service providers, if a woman is receiving financial assistance, it compounds the situation because the higher cost of living is not taken into account when determining monetary support. Although provincial income support is progressive, it can be inadequate to meet basic needs. As was revealed in previous studies (Alberta Council of Women's Shelters, 2019; Thurston et al., 2006; Tutty et al., 2006), barriers, such as a lack of transportation, housing, affordable childcare, educational support, and community resources due to geographic isolation, make IPV circumstances worse and continue to persist. In our study, dire situations for women experiencing IPV in rural and Northern Alberta have at their core the need for more shelters in rural areas, which is consistent with literature that describes homelessness affecting women in Alberta (Alberta Council of Women's Shelters, 2019; Siemieniuk et al., 2013). Without access to second-stage or subsidized housing, there can be no housing for women victims of IPV, as it forces them to return to abusive relationships (De Riviere, 2014).

This study also identified the challenges faced by service providers due to barriers for women to access second-stage or subsidized housing and noted the need for specialized services such as language translators. Similar to a study by Tutty et al. (2005), the lack of IPV-specific education for police, lawyers, victim support counsellors, judges, and Crown prosecutors that result in service providers being ill-prepared to handle the range of IPV situations remains an issue in rural and Northern Alberta. Thus, more training for healthcare professionals, local law authorities, and shelter workers is needed to address IPV. Unfortunately, neglect of professional development (Tutty et al., 2006) may result in the revictimization of women, which includes attitudes of discrimination and the normalization of IPV in the court system experience of women. Gaps in mental health services and programs, in the availability of counsellors, and in accessing therapy groups that respond to the gendered traumatization and adverse childhood events of women experiencing IPV, as well as IPV offenders (Tutty et al., 2006), still exist in rural areas. These challenges are made worse for service providers helping to mitigate and eliminate IPV in rural and Northern Alberta.

To reduce IPV in rural and Northern Alberta, knowledge and action must be drawn together to understand the sociocultural context of IPV, the need for formal services, the leveraging of informal supports, and the harnessing of protective factors in order for service providers to assist women experiencing IPV better and to facilitate transition from an IPV environment to an IPV-free environment. A growing body of literature has suggested people's experiences of and responses to IPV are shaped by key influences associated with traditional culture (O’Campo et al., 2015; White & Satyen, 2015). In addition, hope is described as a strength that exists in rural and northern communities (Faller et al., 2018), along with the desire to come together and meet the challenges of the day. Such community fortitude is important and can be used to intensify an IPV response by service providers and community members that benefit women and all regions. A research and development agenda for IPV preventative models that considers rural and northern geography and integrates sectors such as health, justice, education, social development, media, and entertainment, requires exploration. Future research that examines the implementation of models of IPV prevention in rural and northern communities is essential. An understanding of culturally appropriate education, prevention, and intervention programs that match the target cultural groups, representative of Alberta, is imperative if IPV in rural and northern regions is to be reduced. We also recommend further testing of this framework and application to other rural areas of Canada.

## Limitations

A limitation of this research is that the interview participants included only IPV service providers. The voices of women experiencing IPV in rural and Northern Alberta were not accounted for; as such, only a special knowledge view was included in this research. Future research needs to elicit the perspectives of women who have experienced IPV for comparison. Another limitation is that the sample was restricted to one Canadian province. Last, it is possible that disclosure of specific details by service providers did not reflect an understanding of women in rural and Northern Alberta affected by IPV. It is important to understand that differences exist within the perceptions of service providers working with different cultural groups in diverse geographical areas and with women in varying IPV situations and circumstances. However, as all participants expressed a willing desire to express their perspectives and had declared experience living in rural areas of Alberta, we believe their perspectives are accurate and valid.

## Conclusion

Overall, in their work with women experiencing IPV in rural and Northern Alberta, service providers recommended paying more attention to understanding different sociocultural contexts that require new considerations and responses, actions that facilitate empowerment and resilience in women, and factors that will lessen the magnitude of harm from risks and stressors for women experiencing IPV. First, service providers need to be trained and educated on culturally safe interventions and are calling for more culturally appropriate professional development. Alberta is home to Indigenous, transient, agricultural, and LGBTQ communities. The province has become much more culturally diverse in recent years and has welcomed immigrant and refugee members into rural and northern areas. Second, shelter options that include second-stage housing, followed by affordable housing need to be addressed by local and government representatives so that women in rural and northern regions have access to safe options. These outcomes would be enhanced through collaborative partnerships between service providers and the use of integrated case management. Third, service providers need to share knowledge, resources, and strategies that assist to leverage informal supports for women experiencing IPV. Assisting women in IPV relationships and finding solutions of a social nature requires a shared understanding of the social connections, quality of life, community values, gender equality concerns, and IPV education needs particular to communities in rural and Northern Alberta. Finally, service providers stressed the importance of providing women experiencing IPV in rural and Northern Alberta with knowledge of services and effective ways of accessing them. The facilitation of problem solving by service providers with women would lead to more positive outcomes related to safe shelter challenges as well as concerns associated with transportation, childcare, addiction treatment, specialized sexual assault services, specialized IPV legal support, programs for children exposed to IPV, and other provincial police, victim, and family services.
